# Aerosolized Surfactant for Preterm Infants with Respiratory Distress Syndrome

**DOI:** 10.3390/children8060493

**Published:** 2021-06-10

**Authors:** Mandy Brasher, Thomas M. Raffay, M. Douglas Cunningham, Elie G. Abu Jawdeh

**Affiliations:** 1 Department of Pediatrics/Neonatology, College of Medicine, University of Kentucky, Lexington, KY 40506, USA; doug.cunningham@uky.edu; 2 Department of Pediatrics/Neonatology, College of Medicine, Case Western Reserve University, Cleveland, OH 44106, USA; tmr12@case.edu

**Keywords:** aerosolized surfactant, nebulize, respiratory distress syndrome, preterm infants

## Abstract

Currently, the administration of surfactant to preterm infants with respiratory distress syndrome (RDS) mainly relies on intratracheal instillation; however, there is increasing evidence of aerosolized surfactant being an effective non-invasive strategy. We present a historical narrative spanning sixty years of development of aerosolization systems. We also offer an overview of the pertinent mechanisms needed to create and manage the ideal aerosolization system, with a focus on delivery, distribution, deposition, and dispersion in the context of the human lung. More studies are needed to optimize treatment with aerosolized surfactants, including determination of ideal dosages, nebulizer types, non-invasive interfaces, and breath synchronization. However, the field is rapidly evolving, and widespread clinical use may be achieved in the near future.

## 1. Introduction

Respiratory distress syndrome (RDS) secondary to surfactant deficiency is a common problem and major cause of morbidity and mortality in neonates, especially in preterm infants [1]. Outcomes and management of RDS have improved significantly with the discovery of exogenous surfactants and the refinement of instillation strategies. There are currently multiple natural and synthetic exogenous surfactants with comparable benefits and risks [2]. Intratracheal instillation, in various strategies, remains the most widely accepted method of surfactant administration to infants with RDS [3,4]. However, there is increasing evidence suggesting that supraglottic administration via aerosolization is feasible and effective in preterm infants with RDS [5,6,7,8].

## 2. The Challenging Road towards Successful Clinical Application: Historical Narrative

The clinical success of aerosolized surfactants has been fraught with difficulty. For more than six decades, many attempts to aerosolize an exogenous surface tension reducing agent have been reported. However, within the past decade, multiple reports have demonstrated effective aerosol models for delivering exogenous surfactants in tandem with non-invasive ventilatory support.

The first attempts of an aerosolized surfactant came in a preliminary study in 1964 by Robillard et al., who attempted to administer an aerosol of the synthetic surfactant beta-gamma-dipalmitoyl-L-alpha-lecithin (DPL) to 11 infants with RDS [9]. The eight infants who survived had a modest improvement in their retraction scores. Although these results were not clinically significant, they provided optimism towards aerosolizing surfactants. The second study of this era, Chu et al. in 1967, comprehensively explored an aerosolized DPL via several methods [10]. Using a freon gas carrier, DPL was aerosolized and administered to the excised lungs of 14 infants at autopsy (7 of whom carried the diagnosis of RDS and 7 with normal lungs), with no observed changes to lung elasticity. Subsequently, DPL was aerosolized into the hood of 15 spontaneously breathing live infants. Only half of the infants showed some improvement in their respiratory distress after treatment. In retrospect, this particular form of synthetic surfactant is a rigid crystalline gel at physiologic temperature and also does not have the advantage of surfactant proteins. After these two studies, several decades passed, with advancements in the use of non-invasive respiratory support and instilled surfactant for RDS.

Surfactant, as an aerosol, was revisited by Lewis et al. in 1991 [11]. In an effort to distribute surfactant more uniformly throughout the lungs, Lewis et al. administered aerosolized surfactant to premature lambs for comparison with instilled surfactant. All groups still utilized an endotracheal tube with subsequent ventilator support, and the aerosolized surfactant was released synchronized with inhalation. Lambs treated with aerosolized surfactant demonstrated improvement in their ventilation on par with the instilled surfactant group. Despite this match in clinical outcome, they estimated the dose of aerosolized surfactant deposited in the lungs was only 2 mg/kg. This is markedly less than the instilled surfactant dose of 50 mg/kg, with an estimated 19 mg/kg of surfactant delivered to the lungs.

The promising results of the Lewis et al. lamb study were naturally followed by a proliferation of animal studies, lung cast modeling, and occasional infant feasibility studies. Some studies investigated different nebulizer types, such as Henry et al. in their 1996 study using ultrasonic nebulized surfactant in preterm lambs [12]. While they were able to correlate aerosolized surfactant deposition with better-ventilated regions of lung, the treated lambs did not show improvement in oxygenation. In 1998, Fok et al. also utilized an ultrasonic nebulizer and were able to deliver high levels of surfactant in a rabbit model but found only mediocre clinical improvement [13]. The best explanation for this lack of clinical improvement despite adequate surfactant deposition is surfactant inactivation by the high levels of heat generated by ultrasonic nebulizers.

Unique approaches were attempted to solve the dilemma of delivering effective surfactant to the distal airways, such as the hydroscopic surfactant used in the 1996 rabbit model by Ellyett et al. [14]. This “dried” surfactant would progressively become saturated with water during transit, becoming heavier when in the distal airways to settle there. This was preliminarily successful but not pursued further. In another case, a feasibility study of 20 infants used a specially modified nasopharyngeal tube placed behind the soft palate for jet-nebulized surfactant, with improvement in respiratory outcomes [15].

At the turn of the century, Berggren et al. performed a clinical pilot study in Sweden of nebulized surfactant therapy for neonatal RDS [16]. The study included 34 newborns on continuous positive airway pressure (CPAP) placed into either a treatment group of 480 mg aerosolized poractant alfa via a jet nebulizer or CPAP alone. There were no adverse events noted from aerosolization. However, there was also no documented benefit, with no difference in respiratory outcomes between treatment and control groups, including days on CPAP or need for mechanical ventilation (38% treated vs. 31% controls). Through the 2000s, two new approaches emerged. First, lucinactant, a second-generation synthetic surfactant comprised of two phospholipids, a fatty acid, and a synthetic surfactant protein B (SP-B) peptide replacement, KL_4_, came to the forefront [5,17,18]. Unlike the surfactant proteins in natural surfactants, the synthetic KL_4_ is more resistant to the aerosolization process [19]. The second shift in the aerosolization process manifested as a new focus on vibrating membrane nebulizers. Finer et al. in 2010 presented the combination of prophylactic lucinactant and vibrating membrane nebulizers in a safety and feasibility study of 17 infants [5]. The failure rate, defined as intubation, occurred less frequently in infants receiving aerosolized lucinactant (29%) compared to historical controls.

Coming to the most recent years, deliberate combinations of nebulizers, surfactants, and nasopharyngeal interfaces have emerged as a non-invasive approach to aerosolized surfactants. Minocchieri et al. offered a blinded randomized controlled trial of 64 infants in 2019 using the eFlow neonatal vibrating mesh nebulizer to administer poractant alfa via a dedicated nebulizer adapter [6]. They showed a decrease in intubation rates with a relative risk reduction of 0.57 (95% CI: 0.34 to 0.94). The same year, Sood et al. presented an uncontrolled 17 infant safety and feasibility pilot study using the MiniHeart Lo-Flo jet nebulizer via short binasal prongs; the infants tolerated the treatments well, with clinical improvement, as measured by heart rate, pCO_2_, and blood pH [7]. The most robust example of the customized interface is the 2020 Cummings et al. study, which included 457 infants randomized to standard of care (CPAP) or treatment with aerosolized surfactant via a modified Solarys pneumatically driven nebulizer with the patient interface in a pacifier shape [8]. This multicenter study included both preterm and term infants with respiratory distress on CPAP support, with randomization within the first 12 h of life. The study allowed multiple doses of aerosolized surfactant to be administered as per the clinical team. The primary outcome was endotracheal intubation during the first 4 days of life. There was a decrease in intubation in infants who received surfactant (RR: 0.51 (90% CI: 0.41–0.63)) compared to CPAP alone. The Cummings et al. study is the largest trial that has investigated aerosolized surfactant. More studies, including a systematic review, are currently underway that will provide more insight into the effectiveness of aerosolized surfactant, including prevention of intubation and subsequent mechanical ventilation [20].

## 3. Nebulizers

The evolution and composition of nebulizers play an important role in the success of aerosolized surfactants. There are three general categories of nebulizers, with subsequent subtypes and modifications within each family. This includes jet nebulizers, ultrasonic nebulizers, and mesh nebulizers. Table 1 summarizes the various nebulizers utilized to aerosolize surfactants in clinical studies in infants.

Jet nebulizers, the oldest, most common, and affordable type, use a pressurized gas, the “jet”, that passes through the liquid drug solution and shears droplets from the surface to form the aerosol. There are many modifiable variables to jet nebulizer output, including the density, pressure, and flow rate of the compressed gas, which influence the aerosol particle size as well as variations in the arrangement of tubing and aerosol containment, allowing continuous aerosol flow or medication release during inhalation only. Disadvantages to jet nebulizers include their inefficiency as their reservoir tends to retain a large proportion of medical solution [21,22,23].

Ultrasonic nebulizers use electrical input through a transducer that converts the energy into high-frequency vibrations, creating a standing wave from which aerosolized droplets are produced. In terms of surfactant administration, ultrasonic nebulizers have the major drawback of heating the solution, presumptively leading to the denaturing of proteins, an important component of effective surfactant [21,23].

Mesh nebulizers are the newest nebulizer device type, emerging as the nebulizer of choice for new pharmaceuticals, distinguished by their efficiency [24]. This nebulizer also uses an electric power source to create high-frequency vibrations to force the medication solution through a fine mesh perforated by tapered holes, generating the aerosol. Variations within this nebulizer class are primarily based on the parameters of the mesh: different material composition of the mesh and varying diameters of the tapered hole openings, containing a range (1000–4000) of holes across the mesh surface. The primary disadvantages of this nebulizer type include blockages of the mesh holes, the difficulty of cleaning it, and the higher cost of the machine compared to other nebulizer types [21,22,23].

## 4. Delivery, Deposition, Distribution, and Dispersion of Aerosolized Surfactant

**Figure 1:**
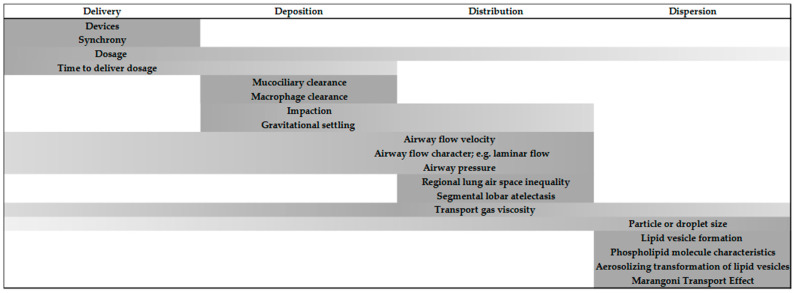
Dynamic processes for the movement of aerosolized surfactant within the human infant lung.

Aerosolized surfactant confronts unique challenges, overcoming deposition in the upper airways and being distributed to the deepest segments of the lung and effectively dispersed within gas-exchanging alveolar tissues. A considerable portion of a delivered therapeutic surfactant dosage is known to be lost before a sufficient amount descends below the alveolar ducts. As little as 1% to 14% of a dosage has been reported to reach the most distal lung parenchyma [25,26]. However, a more recent report using a newborn piglet model introduced atomization rather than nebulization [27]. Their investigational system is a multilumen flexible catheter capable of delivering low airway flow simultaneously with intermittent delivery of surfactant. The median particle size was 40–60 μm. Despite the larger particle size, they demonstrated that 40% of the surfactant is distributed by scintigram in the distal lung regions. They achieved a remarkable supraglottic delivery that overcomes tracheal and large bronchial airway losses to deposition yet provided bilateral lung distribution and alveolar space dispersion. To meet the challenges of surfactant delivery as an aerosol, animal data and in-vitro models have revealed considerable insight into the potential for it to become an effective therapeutic agent. Multiple variables have been determined that provide guidelines for translational considerations in the development and clinical application of aerosolized surfactant for infants (see Figure 1).

### 4.1. Delivery of Aerosolized Surfactant

Aerosol delivery is dependent upon the different designs of the many nebulizing devices currently available (see “Nebulizers” above, Table 1). In turn, for surfactant therapy, the various nebulizers generate an aerosol with unique characteristics that are dependent upon the dosage, synchrony with inspired breaths, and desired time for delivery. An immediate goal of aerosolizing exogenous surfactant is to establish an effective supraglottic delivery system in tandem with existing non-invasive ventilatory support and set the stage for surfactant dose distribution to the multiple lobes of the lung. Recognizing that a major portion of the delivered dose is lost to the deep pharyngeal fossa [25,26], an attempt should be made to synchronize the delivery with a majority of spontaneous breaths. Delivery of an aerosol requires consideration for the time needed to complete a dose. This is true whether the delivery is a continuous application or delivery is in synchrony with either mechanically inspired breaths or spontaneous inspiration. Reports of 20 min to 2 h for completion of aerosol dose delivery leave much to be resolved as to the effectiveness [28,29]. Dosage amount per delivery, in milligrams of a phospholipid, varies from 50 to 200 mg/kg in either animal or human subjects, but all have been shown to improve gas exchange as the ultimate test of aerosolization success [11,30].

### 4.2. Deposition of Aerosolized Surfactant

Much of an aerosolized dosage is lost in the airways due to the velocity of the air stream and the impaction of the droplet against the walls of the trachea and bronchi. Further loss is incurred with gravitational settling upon the ciliated epithelium of the airways [31]. Particles or droplets may be swept proximal by the cilia, or they may be adsorbed in the underlying mucus layer of the ciliated epithelium of the airways. Finally, some dosage is discarded through the clearance action of resident airway macrophages.

### 4.3. Distribution of Aerosolized Surfactant

Aerosol distribution within the regions of the lung may be gravity-dependent or non-gravity-dependent depending upon the position of the infant. Cunha-Goncalves et al. have demonstrated the impact of subject position in a spontaneously breathing piglet model. The lung dose distribution was higher in the prone position. The influence of gravity was also demonstrated, with the highest dose distribution in the lateral decubitus position for the dependent lung [32]. An immediate goal of aerosolization is to reach as much of each lobe as possible with the surfactant dose. In patients with acute respiratory distress, alternating areas of atelectasis will be factors limiting the effectiveness of an aerosol. Asgharian et al. described a system of aerosol generation using a radiolabeled iron chloride solution to identify lung regions and specific lobes in spontaneously breathing adult rats [33]. They demonstrated distribution and dispersion throughout both lungs, with the greatest concentration of the inhalant deep within peripheral lung parenchyma.

Airway flow has been established as an important factor affecting aerosolized surfactants. An optimal airway flow would minimize deposition in the upper airway and enhance distribution to the deepest regions of the lung. At low gas flow, airway turbulence is minimized and laminar gas flow optimized. Laminar airway flow has been shown to enhance particle and droplet distribution [31]. Using catheter-based laboratory models, Syedain et al. [34] demonstrated that with an air jet nebulizer (Microjet™), they could achieve low airway flow and simultaneously generate surface-active small (4 μm) particles/droplets of commonly used surfactant therapeutic agents. Both observations support known optimization of surfactant distribution to the deepest regions of the lung. Moreover, the clinical implications of their observations are that low airway pressures and jet-nebulized aerosol would be compatible with pressure support mechanical ventilation or non-invasive continuous positive pressure ventilation for preterm infants.

### 4.4. Dispersion of Aerosolized Surfactant

An additional goal for achieving adequate delivery of an aerosolized surfactant is to gain sufficient dispersion of the dosage deep within the parenchymal lung tissue, resulting in adequate and sustained gas exchange. To this end, Aramendia et al. offered computational, experimental modeling of a delivery system replicating a preterm infant [35]. Their findings served to confirm that adequate dispersion of aerosolized surfactant at the alveolar level is dependent upon the properties of droplet size, airflow driving pressure, and airflow velocity. A perfluorocarbon was used as a proxy for surfactant, and an intratracheal catheter was used to instill a comparable infant surfactant dosage into a droplet-sizing spectrometer. Their data revealed the ideal droplet size to be 3.97 to 4.08 µm at 4 to 5 atm, approximating earlier work showing the optimum droplet size of 3–4 µm, with slightly reduced size at increased driving pressure [34]. From these aerosol dynamics, droplet size remains paramount in studies for maximal distribution and dispersion of a surfactant dosage within lung tissues [30]. Moreover, Borghardt et al., in a review of the pharmacokinetics of inhaled therapies for respiratory diseases, refer to an aerodynamic droplet size of 0.5–5.0 µm as being critical for the delivery of any “lung-dose”, with the greatest potential to be dispersed at the deepest levels of lung parenchyma [36]. Once a fraction of the aerosolized surfactant has reached the lower airways, nearing the end of the ciliated epithelium, it approaches the alveolar ducts. At this point, the remaining surfactant dosage becomes the lung-dose, ready for transport and dispersal as lipid vesicles within the alveolar ducts and alveolar spaces. The lung-dose becomes one portion that remains as aerosolized droplets and another portion that comes in contact with the air-surface liquid interface. Maximizing the surfactant lung-dose will continue to be a clinical goal as newer nebulizers are developed. Presently, means for monitoring lung-dose effect will rely mainly on effective infant pulmonary gas exchange, but evolving lung ultrasound technology promises to add imaging for evidence of greater surfactant lung-dose distribution and dispersion.

## 5. The Potential Benefit of Aerosolized Surfactant

The major upside of aerosolized surfactant is reducing the need for both intubation and positive pressure ventilation. Adverse events from endotracheal intubations are common in neonates, with incidences ranging from ~20–40% [37,38]. Associated complications include: physiologic changes such as hypoxemia and bradycardia [38,39,40]; oral, airway, and esophageal trauma/injury [37,38,41]; or inappropriate surfactant administration [38,42]. Successful intubation often requires multiple attempts [42,43]; consequently, the higher the number of attempts, the more the risk for adverse events [37], an important consideration with the rising concern for endotracheal intubation proficiency among trainees and providers [44,45,46,47]. In addition, positive pressure ventilation has well documented adverse outcomes, especially in preterm infants [48,49]. Positive pressure ventilation early after birth (even for a few breaths) causes lung injury and blunts the therapeutic effects of the surfactant [50,51]. The initial injury is further exacerbated by continuous and prolonged mechanical ventilation in the neonatal intensive care unit [52,53,54,55,56,57,58]. Other benefits of a nebulized surfactant include avoiding the risks of sedation medications and paralytic agents, often used for intubation in preterm infants [59].

It is unclear if there is a favorable cost–benefit of using aerosolized surfactants. The human studies used high surfactant dosages to account for losses. The duration of administration of aerosolized surfactants is comparable and sometimes longer than surfactant instillation strategies and, currently, not a major advantage. Finally, the recent clinical studies that showed benefit from aerosolized surfactants did not include preterm infants with severe RDS and lacked sufficient numbers of extremely preterm infants, an important consideration for clinical implementation.

## 6. Conclusions

We presented a historical narrative of the progress seen in the application of aerosolized surfactants over the past six decades. There is rising and promising evidence supporting the use of aerosolized surfactant in preterm infants with mild to moderate RDS. However, there is a paucity of data in infants with severe RDS and the smallest extremely preterm infants. This movement of assessing aerosolized surfactants shows no signs of slowing, with multiple ongoing national clinical trials [60,61,62]. We also summarized in this manuscript the mechanisms of ideal aerosolization strategies, with a focus on delivery, distribution, deposition, and dispersion when applied to the human lung. We believe these are important concepts for neonatologists and clinicians to understand as we translate the evidence to clinical practice in the future. Importantly, more studies are needed to optimize dosage, nebulizer types, non-invasive interfaces, and synchronization before supporting wide use in preterm infants. If an ideal surfactant aerosol can be advanced with non-invasive means of ventilatory support and established efficacy and safety for newborn premature infants, a major therapeutic step forward can be achieved in the near future.

## Figures and Tables

**Table 1:** Summary of aerosolized surfactants and nebulizers utilized in clinical studies in preterm infants.

Study	Population (n)	Nebulizer (Type)	Interface	Surfactant	Dose	Flow	Particle Size	Comparison	Outcome
Jorch et al., 1994	28–35 wk GA (20)	Intersurgical RO252/ME (jet)	Modified nasopharyngeal tube placed behind soft palate	Beractant	150 mg/kg per treatment	8 L/min	<4 µm	Pre/post treatment	Significant improvement of A-a gradient, PaCO_2_, and Silverman score
Berggren et al., 2000	27–34 wk GA (34)	Aiolos ^®^ (jet)	Infant Flow System^®^ nasal CPAP	Poractant alfa	480 mg	7 L/min	<2 µm	Aerosolized surfactant vs. CPAP alone	No benefit in treatment group
Finer et al., 2010	28–32 wk GA (17)	Aeroneb Pro ^®^ (vibrating mesh)	Fisher-Paykel nasal prongs	Lucinactant	72 mg per treatment	1 L/min	1.9 ± 0.3 µm	Pre/post treatment Historic control intubation rate	Mean FiO_2_ decreased after treatment. 30–32 wk GA infant intubation rate lower than historic control
Sood et al., 2019	24–36 wk GA (17)	Low Flow MiniHeart (jet)	Short binasal prongs	Beractant	100–200 mg/kg per treatment	2 L/min or less	Not reported	Pre/post treatment	Decrease in heart rate and pCO_2_; increase in pH
Minocchieri et al., 2019	29–33.6 wk GA (64)	eFlow-Neos (vibrating mesh)	Face mask (not specified)	Poractant alfa	200 mg/kg per treatment	6–8 L/min	Not reported	Aerosolized surfactant vs. CPAP alone	Decreased intubation rate in treated 32–33.6 wk GA subgroup
Cummings et al., 2020	23–41 wk GA (457)	Modified Solarys (pneumatically driven)	Custom pacifier mouthpiece	Calfactant	210 mg/kg per treatment	8–10 L/min	4.5 µm	Aerosolized surfactant vs. usual care	Decreased intubation rate in treated infants
